# Genomic analysis of early murine mammary gland development using novel probe-level algorithms

**DOI:** 10.1186/gb-2005-6-2-r20

**Published:** 2005-02-01

**Authors:** Stephen R Master, Alexander J Stoddard, L Charles Bailey, Tien-Chi Pan, Katherine D Dugan, Lewis A Chodosh

**Affiliations:** 1 Department of Cancer Biology, University of Pennsylvania School of Medicine, Philadelphia, PA 19104-6160, USA; 2 Department of Pathology and Laboratory Medicine, University of Pennsylvania School of Medicine, Philadelphia, PA 19104-6160, USA; 3 Department of Medicine, University of Pennsylvania School of Medicine, Philadelphia, PA 19104-6160, USA; 4 Abramson Family Cancer Research Institute, University of Pennsylvania School of Medicine, Philadelphia, PA 19104-6160, USA; 5 Department of Pediatrics, Children's Hospital of Philadelphia, Philadelphia, PA 19104, USA

## Abstract

We describe a novel algorithm (ChipStat) for detecting gene-expression changes utilizing probe-level comparisons of replicate Affymetrix oligonucleotide microarray data. A combined detection approach is shown to yield greater sensitivity than a number of widely used methodologies including SAM, dChip and logit-T. Using this approach, we identify alterations in functional pathways during murine neonatal-pubertal mammary development that include the coordinate upregulation of major urinary proteins and the downregulation of loci exhibiting reciprocal imprinting.

## Abstract 2

A novel algorithm (ChipStat) is presented for detecting gene-expression changes from Affymetrix microarray data. The method is used to identify changes in murine mammary development.

## Background

The widespread use of DNA microarrays to measure transcript abundance from a significant fraction of the genome has proven to be a valuable tool for identifying functional cellular pathways as well as for capturing the global state of a biological system [1-4]. These arrays have typically been constructed by spotting large, pre-synthesized strands of nucleic acid on an appropriate surface [5] or by directly synthesizing smaller oligonucleotides *in situ *at defined locations [6]. The latter technique has been implemented in Affymetrix oligonucleotide microarrays designed for expression analysis. Because hybridization to short (25-mer) oligonucleotides is used to measure expression, Affymetrix arrays contain multiple, independent oligonucleotides designed to bind a unique transcript. In this way, specificity and a high signal-to-noise ratio can be maintained despite the noise due to the hybridization itself. When the intensity of hybridization to a given oligonucleotide designed to detect the transcript (a 'perfect match' probe, PM) is corrected by its corresponding (single base-pair 'mismatch', MM) control, an estimate of gene expression (PM - MM) is derived. This probe pair value is then combined with values from the other, independent, oligonucleotides designed to bind the same transcript (together designated the probe set) to obtain a more robust estimate of transcript abundance [7].

The ability to sensitively detect changes in gene expression is crucial for a transcript-level analysis of developmental processes and other processes involving changes in the relative sizes of cellular compartments. Early attempts to limit the false-positive rate of microarray studies focused on the magnitude of fold-change in gene expression (see, for example [1]). For studying purified cell populations, where a substantial change in gene expression is more likely to reflect biologically relevant function, such a crude limitation was acceptable. However, adequate studies of complex tissues require a substantially more sensitive method of detection. For example, a small yet reproducible change in gene expression within a whole organ may reflect a substantial expansion or regulatory change within a subpopulation of cells that overexpress a given gene relative to the surrounding tissue. Thus, a method for identifying such small, statistically significant changes in gene expression is required.

Because of the variety of techniques used to measure gene expression, it has become commonplace to utilize simple, numerical estimates of gene expression as the starting point for such identification. One major drawback to this approach has been that individual probe cell information from Affymetrix microarrays is routinely discarded. This issue has only recently begun to be addressed [8-10], and it appears that a substantial amount of useful information can be obtained from probe-level analysis.

An additional compromise has been driven by the practical difficulties of performing large numbers of microarray experiments. Given limited samples, permutation of the existing experimental dataset, rather than use of independent sets of control samples, has been widely used to estimate the statistical significance of differential gene expression [11]. Although this technique has been useful given the historically high cost of performing microarray analysis, it may inherently limit the sensitivity of the results obtained. As such, a test for differential gene expression that utilizes a 'gold standard' negative-control dataset would have clear advantages.

The impetus for the work described here is the desire to sensitively identify coherent patterns of gene expression during mammary gland development. At 2 weeks of age, the female FVB mouse mammary gland exists as a rudimentary epithelial tree embedded at one end of a fat pad composed of adipose tissue and fibroblasts. Previous work has demonstrated a fundamental transition in the composition of the mammary adipose compartment from brown fat to white fat during early development [4]. By 3 weeks of age, the onset of puberty heralds the beginning of the process of ductal morphogenesis, which results in the formation of the branching epithelial tree of the adult gland. The onset of puberty results not only in the rapid growth of a ductal epithelial tree but also the appearance of specialized, highly proliferative structures known as terminal end buds that elaborate this tree via branching morphogenesis [12,13]. Furthermore, puberty is known to be a time of increased susceptibility to carcinogenesis [14,15]. Thus, a detailed examination of transcriptional changes during this period would be of substantial use.

We describe here a novel algorithm for sensitively detecting gene-expression changes using information derived from individual probe cell hybridizations to Affymetrix oligonucleotide microarrays. In addition to modeling the predicted behavior of this algorithm, we have generated an independent cohort of control samples derived from the murine mammary gland that can be used to empirically calibrate its statistical behavior. We have then used this algorithm to analyze a biological transition in early murine mammary gland development in order to compare the sensitivity of this approach to other commonly used algorithms. In conjunction with a second novel algorithm, we have developed an aggregate approach to the reliable detection of differential gene expression that yields substantially improved sensitivity across a range of false-positive rates and have applied this approach to the analysis of early murine mammary gland development.

## Results

A variety of traditional statistical methods, such as the *t *test, have been used in conjunction with microarray datasets to detect changes in gene expression (see for example [16]). Given the large numbers of genes tested, it is widely recognized that a stringent threshold for statistical significance is necessary in order to reduce the number of false positive changes. For example, a threshold of statistical significance of *P *< 0.001 would be expected to yield around 100 false positives on a typical array measuring 10,000 genes. Some algorithms, such as significance analysis for microarrays (SAM) [11], explicitly control the number of expected false-positive results using permutations of the existing dataset. Regardless of the method utilized, statistical differences are typically calculated on the basis of an aggregate measure of gene expression (a gene signal). However, a fundamental difficulty with these methods is that they often do not have the requisite statistical power to sensitively detect changes in gene expression after correction for multiple hypothesis testing. We reasoned that utilizing the multiple hybridizations to independent oligonucleotides on the Affymetrix platform might allow us to develop a method for detecting expression changes with substantially greater statistical power.

To test this approach, we developed a novel analytical algorithm that is based on identifying individual differences at a given statistical significance between corresponding probe pairs. To a first approximation, the signal on any given probe cell can be modeled as:

S = M + E(b) + E(p) + E(h), E ~ N

Where S is the signal detected on the microarray, M is the average message level in a given experimental state, E(b) is noise due to biological variation between animals or animal pools, E(p) is the noise due to variations in sample measurement, and E(h) is the noise inherent in hybridization to oligonucleotide features on the array. The goal of our analysis was to identify a method that would allow us to reliably distinguish significant differences in M under particular experimental conditions.

Given this model, we reasoned that the relative magnitude of E(b) + E(p) (the experimental noise) compared with E(h) (the hybridization noise) should determine whether comparisons between individual probe pairs would be useful. If the bulk of noise in our microarray data was due to factors influencing the level of transcript available for measurement (that is, E(b) + E(p) >> E(h)), then individual probe-pair measurements should only reflect the pre-hybridization bias in transcript availability. In this case, the *t*-test or other measurement based on the average of the probe set would be expected to perform as well as an algorithm based on individual probe-pair comparisons. In contrast, if most noise in the measurement of true transcript level exists at the level of hybridization to a given oligonuclotide (E(b) + E(p) << E(h)), then the independent measurements of probe-pair differences more closely approximate independent measurements of differences in gene expression. In the most extreme case - if E(h) is sufficiently larger than E(b) + E(p) - each oligonucleotide in the probe set could be considered as an independent measurement of gene expression and the probability of observing a given number of probe pairs changing under the null hypothesis would be determined by the binomial distribution.

**Figure 1:**
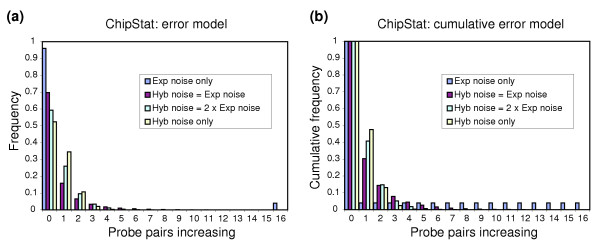
ChipStat behavior using simulated biological/experimental + hybridization noise model. The behavior of the ChipStat algorithm was evaluated (*p*_ps _= 0.05, 16 probe pairs per probe set) using a Monte Carlo model in which the ratio of biological + experimental noise (E(b) + E(p)) to hybridization noise (E(h)) is constant (see text for further details). Results are shown for E(h) = 0 (Exp noise only; blue), E(h) = E(b) + E(p) (Hyb noise = Exp noise; red), E(h) = 2 × (E(b) + E(p)) (Hyb noise = 2 × Exp noise; green), and E(b) + E(p) = 0 (Hyb noise only; yellow). The total number of probe sets simulated (11,820) was chosen to match the number of probe sets containing 16 probe pairs per probe set on the Affymetrix MG_U74Av2 array. The number of probe pairs increasing by chance is shown on the *x *axis, and the fraction of total probe sets simulated is shown on the *y *axis. This simulation was repeated 100×, and the average of these results is shown. (**a**) Probability of the indicated number of probe pairs increasing. (**b**) Cumulative *P *value (equal to or greater than the indicated number of probe pairs changing).

To explore this possibility, we implemented an algorithm, hereafter designated ChipStat, that takes corresponding probe pairs across two comparison groups and tests them for statistical significance with *P *less than a fixed value (hereafter denoted *p*_ps_). To avoid making assumptions about equal variance in both groups, a heteroscedastic *t*-test is used. We would expect that probe sets in which larger numbers of individual probe pairs show a significant change in the same direction are more likely to be measuring differentially regulated genes. Thus, for any given probe set, the number of probe pairs (0-16) changing in a given direction with *P *less than *p*_ps _is tabulated and used as a measure of the significance of change in gene expression. We simulated the expected behavior of this algorithm under the null hypothesis (no difference in gene expression) across various ratios of E(b) + E(p) and E(h) (see Materials and methods for details). Results are shown in Figure 1.

### Validation and optimization of the ChipStat algorithm

Although this approach provides a statistical methodology for identifying changes in gene expression, it is only possible to directly calculate a *P *value associated with this change in limiting cases. If E(h) >> E(b) + E(p), the binomial distribution can be used to calculate the resulting significance (given the number of changes, total number of probe pairs, and *p*_ps_); however, the relative contributions of E(h), E(b), and E(p) to the total error function are not known *a priori*.

**Figure 2:**
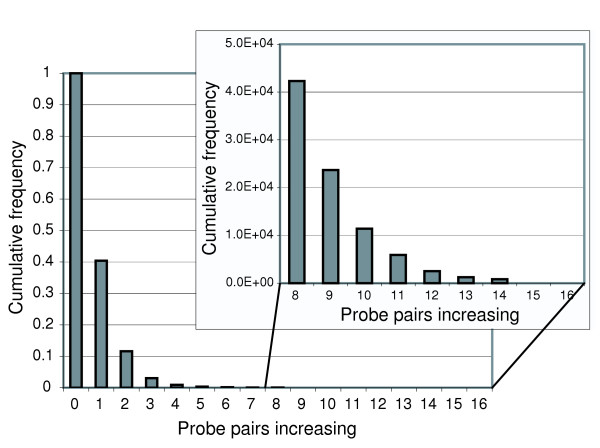
Empirical measurement of the ChipStat null distribution. Mammary gland tissue was harvested from six separate, biologically identical pools of FVB (MTB) mice, and hybridization data to Affymetrix MG_U74Av2 microarrays was obtained. Comparisons of all possible three versus three combinations (total 20) were performed using ChipStat (*p*_ps _= 0.05), and the number of significant increases was tabulated for all probe sets containing 16 probe pairs per probe set (total = 11,820). The cumulative average probability is shown as a function of the number of probe pairs that increase within the probe set.

To empirically measure the null distribution for three-sample versus three-sample comparisons, a cohort of independent control samples for our experimental system was generated. To do this, the third, fourth and fifth mammary glands were harvested from 18 age-matched 5-week-old control female mice. After extraction of RNA, groups of three animals were pooled to create six initial RNA samples. Biotinylated cRNA was then independently prepared from these pooled RNA samples and hybridized to Affymetrix MG_U74Av2 oligonucleotide microarrays, yielding six datasets. All possible three by three combinations were compared across 11,820 probe sets (corresponding to all probe sets on the MG_U74Av2 that contain exactly 16 probe pairs), and the cumulative distribution of false positives as a function of *p*_ps _and the number of probe pairs changed was tabulated. Results are shown for *p*_ps _= 0.05 (Figure 2). It is notable that very few false positives are associated with large numbers (more than 10/16) of probe pairs changing. While the number of false-positive probe sets does not decline as rapidly as the binomial distribution, the overall curve is consistent with a large component of hybridization noise (compare Figures 1 and 2), suggesting the utility of a probe-level approach. Likelihood maximization of our initial statistical model (E ~ N, ignoring probe-specific effects) using results for low numbers of probe pairs (0 to 6) changing suggests that E(h) (hybridization noise) is approximately 2.5 times greater than E(b) + E(p) (experimental noise). We note, however, that the empirically derived null distribution can be used to derive a valid test of significance for ChipStat regardless of the validity of the underlying model and without any direct calculation of relative noise contributions by E(h), E(b) and E(p).

An ideal method for identifying differentially regulated genes would maximize the number of genes identified while maintaining a low fixed number of expected false positives. We have previously shown the utility of testing the statistical overlap of discrete gene lists with biologically relevant annotation in order to identify functional pathways during murine mammary gland development [4]. This maximization is therefore of particular experimental interest. To evaluate the ChipStat algorithm from this perspective, we performed triplicate microarray measurements of RNA derived from the mammary glands of independent pools (more than 10 animals per pool) of wild-type female FVB mice harvested at 2 or 5 weeks of postnatal development. We wished to determine the number of statistically significant increases in gene expression from 2 to 5 weeks of age, a period of postnatal development that encompasses the rapid epithelial proliferation that accompanies ductal morphogenesis in the mammary gland at the onset of puberty [17].

**Figure 3:**
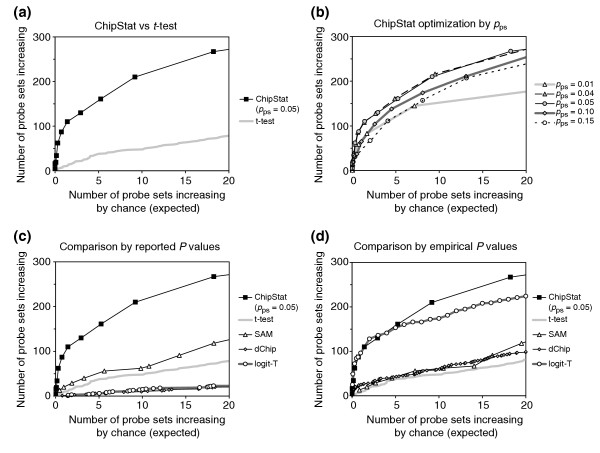
Relative detection sensitivity of differential gene expression. The number of probe sets shown to increase from 2 to 5 weeks of murine mammary gland development was tabulated as a function of the number of probe sets expected to increase by chance. (**a**) ChipStat (*p*_ps _= 0.05), vs *t*-test. (**b**) Optimization of ChipStat sensitivity as a function of *p*_ps_. (**c**) ChipStat vs other techniques: reported *P *values. For ChipStat, the number of probe sets expected to increase by chance was empirically estimated from negative control data. For the *t*-test, SAM, dChip and logit-T, reported *P *values from the 2-week vs 5-week mammary gland comparison were used. (**d**) ChipStat vs other techniques: empirical *P *values. The number of probe sets expected to increase by chance was empirically estimated for ChipStat, *t*-test, SAM, dChip and logit-T (representative points).

ChipStat was used to analyze differences between the 2- and 5-week mammary gland samples (*p*_ps _= 0.05), and the number of statistically significant increases was measured as a function of the number of genes expected to appear on the list by chance. Results are shown in Figure 3a. The number of expected false positives was empirically obtained from the negative-control dataset described previously. Thus, for example, under conditions *p*_ps _= 0.05 with 8/16 probe pairs increasing, where around five genes are expected to be identified by chance, we find that the measured number of differentially regulated genes is around 160. This corresponds to a false-positive rate of approximately 3% (or, conversely, a true-positive rate of approximately 97%). It is also apparent (Figure 3a) that the sensitivity of detection can be 'tuned' on the basis of the number of false positives that are deemed acceptable.

To determine whether the sensitivity of this algorithm could be further optimized, similar analyses were performed at various values of *p*_ps _(Figure 3b). These data suggest that relative sensitivity as a function of false-positive rate is maximized at *p*_ps _approximately equal to 0.04-0.05 (note the similarity of these curves in Figure 3b). Furthermore, while certain other values of *p*_ps _yield increased sensitivity at specific points (for example, *p*_ps _= 0.03 at around four genes expected by chance; data not shown), values of 0.04-0.05 appear appropriate across most highly-significant *P *values. A marked decrease in sensitivity for a given false-positive rate is noted both at low (0.01) and high (0.1, 0.15) values of *p*_ps_.

Although the use of negative-control samples provides a definitive method for evaluating the behavior of our statistical algorithms, we independently verified these results using northern blot hybridization. Genes differentially expressed (6/16 probe pairs increasing, *p*_ps _= 0.04) from 2 to 5 weeks of mammary gland development were identified, and analysis of the control data suggested that fewer than 10 increases would be expected by chance at this significance level (corresponding to *P *< 7.7 × 10^-4^). Manual inspection of the resulting list revealed the presence of a number of genes known to be upregulated during this developmental transition, including cytokeratin 19 (*Krt1-19*), cytokeratin 8 (*Krt2-8*), and *κ *casein (*Csnk*). However, to avoid bias toward previously studied genes or known genes with high fold change, genes were randomly selected from subsets of this list corresponding to high-stringency (*P *< 2.2 × 10^-4^), low-stringency with high fold change (2.2 × 10^-4 ^<*P *< 7.7 × 10^-4^, ≥ 1.8-fold change), and low-stringency with low fold change (2.2 × 10^-4 ^<*P *< 7.7 × 10^-4^, < 1.8-fold change). Results from northern blot analyses using probes for these randomly selected genes are shown in Table 1. Of nine genes selected, eight were shown to change significantly via northern blot analysis.

Of note, the single gene that did not show a significant change (*Ldh1*) was from the low-stringency group and was predicted to show only a 1.37-fold change. In contrast, northern hybridization confirmed the differential expression of other genes with only modest fold-changes (for example, *Sqstm1*, 1.48-fold change from 2 to 5 weeks). As the genes tested were not biased toward higher fold change (only 2/75 genes with fold change > 3 were randomly selected for northern confirmation), our data demonstrate the ability of ChipStat to reliably detect the types of small, reproducible changes in gene expression that are necessary for whole-organ analysis.

### Comparison of ChipStat with other analytical methods

Other methods of detecting differential gene expression have been widely utilized, including SAM [11] and dChip [8]. As previously discussed, SAM utilizes an aggregate (probe-set-level) estimate of gene expression as its analytical starting point. Similarly, although dChip utilizes probe-cell-level analysis to determine the level and statistical bounds of gene expression, it does not explicitly make use of probe-level comparisons for identifying differentially regulated genes. More recently, the logit-T algorithm, which in contrast to SAM and dChip utilizes probe-pair-level comparisons for statistical testing, has been shown to improve differential expression testing performance in a variety of Latin square datasets reflecting technical replicates of samples with spiked-in transcripts [10]. We therefore wished to determine the performance of the ChipStat algorithm relative to these methodologies. Further, as our control dataset incorporates biological and experimental variability in addition to sample preparation and hybridization noise, we reasoned that it would provide a more appropriate estimate of the performance of these algorithms when analyzing data from an experimentally plausible animal model.

SAM, dChip, the *t*-test and logit-T all provide a *P *value estimating statistical significance in the absence of an empirical measurement of the underlying null distribution; Figure 3c shows a comparison with ChipStat when using these estimated *P *values. However, as ChipStat requires the additional information provided by this empirical distribution for statistical calibration, the inherent performance of other algorithms may be underestimated if they are not similarly calibrated. To correct for this difference, the significance of SAM, dChip and logit-T values were assessed using all three by three combinations of the null dataset (given the permutation-based calibration of false-discovery rate utilized by SAM, note that SAM values are not predicted to improve significantly using this method of calibration). Results are shown in Figure 3d. In the case of the *t*-test, results obtained using calculated *P *values are generally within 5% of comparable results using empirically calibrated *P *values. Logit-T and dChip appear much less sensitive when using reported *P *values, although both of these techniques show improvement when calibrated using the control dataset. Of particular note, logit-T performs only slightly less well than ChipStat when calibrated against our control distribution, consistent with the fact that it was the only other algorithm considered that performs probe-pair-level comparisons when testing for differential gene expression.

### Design and validation of the Intersector algorithm

Although the Affymetrix Microarray Suite (MAS) software utilizes probe-level information in identifying differentially expressed genes, its use has been restricted to single-array comparisons. As a result, it has been widely recognized that this approach generates an unacceptably high number of false-positive results. The use of replicate samples, however, might be expected to lower the false-positive rate while achieving a higher sensitivity. We therefore combined pairwise comparisons between triplicate data points in two different groups (that is, nine comparisons in total) and determined differential expression based on the Affymetrix call (for example, increases + marginal increases) for these comparisons. A similar technique, in which a simple majority cutoff (5/9 changes) was considered to denote significant change, has recently been described [18]. Although this approach involves N^2 ^comparisons in general for equal groups of N arrays, it is easily feasible for three-sample versus three-sample comparisons. We have designated this approach Intersector. Significantly, the control data previously generated to calibrate ChipStat also allow us to determine the empirical false-positive rate for Intersector as a function of the number of 'increase' calls and to perform direct comparisons with other algorithms.

**Figure 4:**
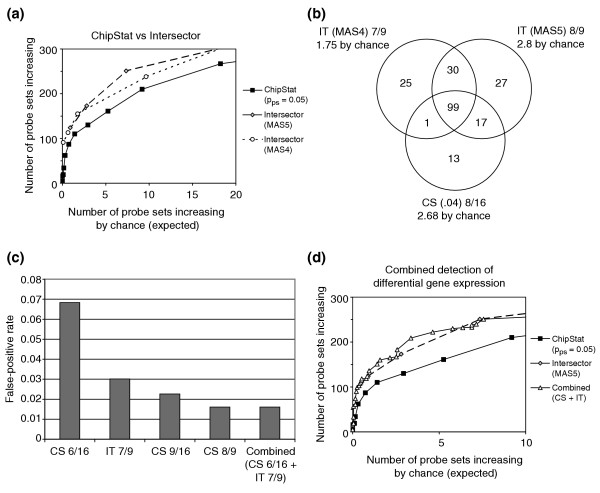
Intersector and ChipStat performance. (**a**) The number of probe sets shown to increase from 2 to 5 weeks of murine mammary gland development was tabulated as a function of the number of probe sets expected to increase by chance, and a comparison of ChipStat (*p*_ps _= 0.05), Intersector (MAS 5.0 change calls), and Intersector (MAS 4.0 change calls) is shown. (**b**) Venn diagram showing distinct probe sets identified by ChipStat and Intersector. The number of genes shown to be differentially expressed at the indicated expected false-positive levels is shown for ChipStat (CS) (*p*_ps _= 0.04), Intersector (IT) with MAS 5.0 calls, and Intersector (IT) with MAS 4.0 calls. (**c**) False-positive rates for ChipStat (CS 6/16: *p*_ps _= 0.05, 6/16 probe pairs increasing; CS 9/16: *p*_ps _= 0.05, 9/16 probe pairs increasing), Intersector (MAS5) (IT 7/9: 7/9 increases or marginal increases; IT 8/9: 8/9 increases or marginal increases), or ChipStat and Intersector together (Combined: intersection of CS 6/16 and IT 7/9) are shown. (**d**) Combined performance of ChipStat and Intersector. Increases from 2 to 5 weeks of mammary gland development are shown for ChipStat alone (*p*_ps _= 0.05), Intersector alone (MAS 5.0), and optimized intersections of ChipStat and Intersector (see Additional data file 1).

The performance of the Intersector algorithm in comparing 2- versus 5-week mammary gland gene expression is shown in Figure 4a. Interestingly, the Intersector algorithm is able to achieve a slightly improved sensitivity at a given false-positive rate when compared with ChipStat. To determine whether the particular version of the MAS algorithm influences this result, all analyses were run using difference calls from both MAS 4.0 and MAS 5.0 (see Figure 4a). Although the number of changes required to achieve similar sensitivity was different, the Intersector results from MAS 4.0 and MAS 5.0 are comparable at a given false-positive rate.

Given substantial differences between the types of probe-pair comparisons performed by ChipStat and MAS, we next wished to ascertain if these algorithms identify the same sets of upregulated genes. Direct comparison requires that the analyses result in comparable false-detection rates. We therefore compared the lists at thresholds corresponding to approximately 2.5 genes expected by chance, and the closest available threshold with each algorithm was chosen. The resulting thresholds were Intersector (MAS4) 7/9 (1.75 expected by chance), Intersector (MAS5) 8/9 (2.8 expected by chance), and ChipStat (.04) 8/16 (2.68 expected by chance). Notably, examination of these lists demonstrates that each algorithm (Intersector with MAS 4.0 data, Intersector with MAS 5.0 data and ChipStat) detects a discrete set of genes that are not detected by the others (Figure 4b). This is particularly intriguing since empirically estimated false positive rates suggest that these groups of genes are not likely to reflect chance fluctuations alone. Thus, in addition to identifying a core set of regulated genes, the Intersector and ChipStat algorithms each detect sets of complementary, nonoverlapping genes that change significantly.

To confirm this result, five out of the 13 genes uniquely identified by ChipStat were randomly chosen for confirmation. One of these genes was undetectable by northern blot hybridization, and the remaining 4/4 showed differential expression in the predicted direction (5 weeks > 2 weeks) (Table 1, and data not shown). This demonstrates that, at comparable levels of statistical stringency, ChipStat correctly identifies differentially expressed genes that are not identified by Intersector. Further, having directly tested approximately 40% of all genes in this category, no false positives were identified. Examination of lower stringency lists (9.5 expected by chance from ChipStat, 7.4 expected by chance from Intersector using MAS5) also revealed sets of genes identified by ChipStat or Intersector alone. For example, the 'Intersector only' list created at this lower stringency contains *α*-, *β*-, and *γ*-casein; previous work in our lab has demonstrated that these genes are differentially regulated with expression at 5 weeks greater than that at 2 weeks (data not shown).

### Development of a hybrid approach

Given the presence of genes uniquely identified by Intersector or ChipStat at a given false positive rate and the feasibility of performing Intersector analysis on small numbers of replicates, we next explored whether a combination of these approaches could further improve overall detection. To test this, all possible pairwise threshold combinations of ChipStat (p_ps _= 0.05, 0/16 to 16/16 probe pairs changing) and Intersector (0/9 to 9/9 increases or marginal increases) were combined, and aggregate lists of genes identified by both algorithms were tabulated (see Additional data file 1). The results demonstrate that a combination of these two approaches can lower the expected false positive rate while maintaining a high sensitivity. For example, the combination of ChipStat (p_ps _= 0.05, 6/16 probe pairs increasing) and Intersector (7/9 increases + marginal increases) detects 209 increasing probe sets with only 3.4 expected to increase by chance (expected false-positive rate less than 2%). A comparison of the false-positive rates for single (ChipStat or Intersector alone) and combined (ChipStat and Intersector) approaches is shown in Figure 4c. Note that the total number of probe sets detected by the combined approach shown in Figure 4c is greater than the number detected by the single approach with a comparable false-detection rate (209 probe sets and 173 probe sets, respectively). The behavior of optimal combinations with respect to the number of genes detected is shown in Figure 4d.

One additional feature of this combined approach is the ability to 'fine-tune' the number of expected false positives. That is, while Intersector (MAS5) allows no choice between approximately three and approximately seven expected false positives (2.8 and 7.35, corresponding to 8/9 or 7/9 changes, respectively), the combined approach provides a smoother continuum of values. More important, these data show that, for certain targeted numbers of expected false positives, a combination of ChipStat and Intersector can provide improved performance in gene detection compared with either algorithm alone.

### Genomic characterization of early mammary gland development

The goal of these methodological developments has been the elucidation of biological mechanisms underlying mammary gland development and carcinogenesis. We therefore used the hybrid ChipStat/Intersector lists representing early mammary gland development as a basis for further exploration of developmental processes during this time period. A complete list of genes differentially expressed between 2- and 5-week murine mammary gland was compiled using the techniques described above. The results are listed in Additional data file 2.

**Figure 5:**
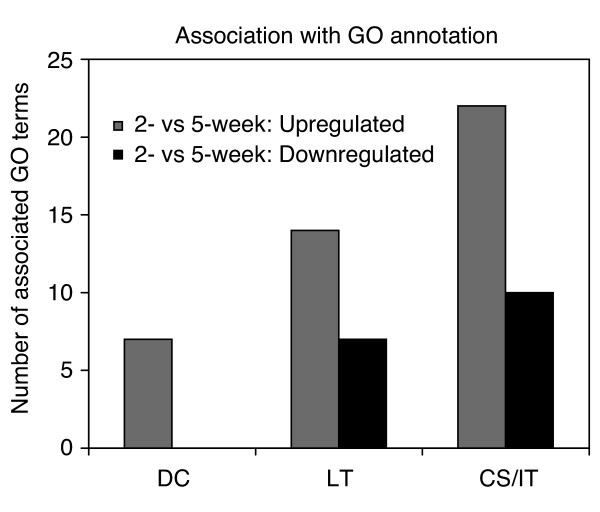
Quantitative association with GO categories. The number of GO terms found to be statistically associated (*P *< 0.05 using within-system bootstrap to account for multiple testing) with lists of differentially regulated genes (2 vs 5 weeks of murine mammary gland development) is shown. Lists of up- and downregulated genes were generated using dChip (DC), logit-T (LT) and a ChipStat/Intersector hybrid (CS/IT) that were matched in stringency to give equivalent numbers of expected false-positive genes.

To identify coherent functional patterns of gene expression during neonatal development through the onset of puberty, statistically significant associations between Gene Ontology (GO) categories [19] and lists of up- and downregulated genes were identified using EASE [20]. Multiple testing correction was performed using within-system bootstrapping, and a corrected significance threshold of *P *less than 0.05 was used. Results are shown in Table 2. Upregulated genes were associated with a total of 22 GO categories, and downregulated genes with 10 categories. In addition, this approach provides a convenient test of whether the increased sensitivity of ChipStat/Intersector yields corresponding power in identifying patterns of biological activity. To test this directly, lists of differentially expressed genes with the same number of expected false positives (empirically calibrated as previously) were identified using dChip and logit-T. These lists were then tested for association with GO annotation, and the results are shown (Table 1, Figure 5). Of note, ChipStat/Intersector lists were associated with a greater number of GO categories than were dChip or logit-T, and this was true for both up- and downregulated gene lists. Consistent with our suggestion that logit-T should be most similar to ChipStat/Intersector because of its use of probe-pair-level comparisons, logit-T also generated lists that are statistically associated with a larger number of GO categories than did dChip (Figure 5), although it did not outperform ChipStat/Intersector. ChipStat/Intersector identified 22/22 of categories associated with any of the list of upregulated genes and 10/11 categories identified using any of the lists of downregulated genes. A single downregulated category ('cellular component: extracellular') was associated only with the logit-T list.

To provide a crude check on the reliability of these results in addition to the confirmation previously performed, gene lists were examined for association with previously described biological processes. In addition to individual genes that are consistent with epithelial proliferation and differentiation (discussed above), several statistically associated categories represent pathways that have been previously described in the mammary gland during this developmental window [4]. These include 'blood vessel development' and 'mitochondrial inner membrane'. The latter category reflects the previously reported decrease in brown adipose tissue at the end of the neonatal period and the corresponding decrease in the capability of the mouse to utilize adaptive thermogenesis to maintain body temperature. Brown adipose tissue is not only rich in mitochondria, but the fatty-acid metabolic pathways necessary for adequate thermogenic activity are also spatially localized at the inner mitochondrial membrane. Of note, this category only reached statistical significance using the ChipStat/Intersector list.

Interestingly, 'pheromone binding' and 'odorant binding' categories are also associated with upregulated expression at the onset of puberty. Genes within these categories are primarily members of the major urinary protein (MUP) gene family, and MUP transcripts (*Mup1*, *Mup3*, *Mup4*, *Mup5*) account for four of the five most highly upregulated genes from 2 to 5 weeks. Large quantities of MUPs are synthesized in the male liver and excreted in the urine, where they bind pheromone and play a role in signaling for complex behavioral traits [21,22]. MUP levels are upregulated during puberty in the liver, although expression levels are much higher in males than in females. While MUP expression within the mammary gland has previously been reported [23,24], its expression was considered to be detectable only with the onset of pregnancy. Our data show that MUPs are highly upregulated in the female mammary gland during the 2- to 5-week transition. Interestingly, *Slp *(sex-limited protein), which also shows sex-restricted expression in the male liver and - like *Mup *expression - is normally repressed by *Rsl *[25], is also significantly upregulated during this period.

Additional examination of these gene lists revealed an interesting transcriptional pattern that is not reflected in the current GO hierarchy. The nontranslated RNA transcript *Meg3/Gtl2 *is significantly downregulated from 2 to 5 weeks of development, and its reciprocally imprinted neighbor *Dlk1 *[26] shows a similar decrease. This is noteworthy because two other genes with decreasing expression, *H19 *(nontranslated RNA) and *Igf2*, are also reciprocally imprinted neighbors, suggesting the possibility of a common regulatory mechanism for altering expression from loci exhibiting this genomic organizational structure (see [27]).

## Discussion

The ability to reliably detect changes in gene expression is critical for the analysis of experimental microarray data. This problem assumes particular importance when analyzing complex mixtures of cells, such as those derived from a whole organ during ontogeny. The challenge can be most clearly seen by considering a small subpopulation of cells that demonstrate a marked change in gene expression. If the expression of this gene is uniform and low throughout the rest of the tissue, the biologically relevant change within a few cells will appear as a low fold change in organ-wide gene expression. A variety of such nonabundant yet developmentally critical cell types have been described. For example, the proliferative capacity of small structures in the mammary gland known as terminal end buds gives rise to the extensive ductal structure that is elaborated during puberty [17]. More recently, the characteristics of mammary stem cells have been described, and these cells have been suggested to serve as targets for carcinogenesis [28,29]. To facilitate the study of such subpopulations within a whole-organ context, therefore, we have developed a novel approach to the analysis of Affymetrix oligonucleotide microarray data.

A variety of nonparametric and parametric statistical tests, including variants of Student's *t*-test, have been used to identify significant changes in gene expression using replicate microarray data. Given the substantial economic investment required for large microarray experiments, attempts have also been made to improve detection of differentially regulated genes through better estimates of the null distribution using permutation analysis; the use of software incorporating such methods, such as SAM [11], has become widespread. A different approach to improved detection (dChip, see [8]) has attempted to use probe-level information to derive an improved estimate of relative gene expression before assessing differential regulation.

While much work has focused on such use of probe-level analysis for estimating gene expression [8,9], the analysis of replicate data at the probe level for identifying differentially expressed genes has only recently become a focus [10,30]. In particular, if hybridization noise contributes a substantial portion of the overall noise inherent in microarray measurements, the use of multiple probe pairs devoted to measuring a single gene suggests a potential approach to overcoming this noise.

The ChipStat algorithm uses heteroscedastic *t*-test comparisons between probe pairs, and the number of probe pairs that change greater than a significance threshold are tabulated. A greater number of consistently changing probe pairs should indicate that the difference is less likely to be due to hybridization noise, and thus this number relates the overall probability that the probe set is measuring a true change in gene expression. The processing time for the ChipStat algorithm scales as a linear function of the number of replicates processed (*O*(N)), and thus it is feasible to apply this approach to much larger numbers of samples.

To assess the statistical significance of ChipStat results, it was necessary to empirically measure the underlying null distribution. While the recent availability of a number of publicly available Latin square datasets representing measurements of spiked-in control samples has greatly facilitated measurements of this sort [31], these datasets reflect technical replicates without biological noise. As we have demonstrated, the behavior of the ChipStat algorithm would be expected to change depending on the relative contributions of biological/experimental noise and probe-level hybridization noise. Thus, a set of negative control samples reflecting an experimental system that include biological noise was required.

To generate these samples, mammary glands from six independent cohorts of mice were harvested. These data provide a true, gold-standard negative control within a representative mammalian experimental system, and we anticipate that their public availability will be similarly useful to the broader scientific community in analytical development and validation. Furthermore, the use of this dataset as an empirical calibration control for ChipStat argues that these results will be valid independent of the adequacy of the statistical noise model used. It is worth noting that the use of pooled groups of animals is likely an important parameter, as single-animals groups, for example, would be expected to exhibit increased biological variability and thus decrease the proportional contribution of hybridization noise.

Given empirical measurements of the expected number of false positives for a given set of analytical parameters, it was possible to assess the relative sensitivity of a variety of algorithms using a positive control dataset (2-week versus 5-week murine mammary gland) known to contain a substantial number of increasing transcripts. Consistent with our hypothesis that probe-level comparison analysis should improve sensitivity, ChipStat was able to substantially outperform a variety of methods (*t*-test, SAM, and dChip) based on aggregate gene-expression measures (Figures 3c,d). Furthermore, this remained the case even when the statistical significance of dChip was recalibrated using a negative control dataset.

Recently, Lemon *et al*. have described a method (logit-T) that is also based on probe-level *t*-test comparisons for identifying differentially expressed genes [10]. The logit-T algorithm estimates statistical significance using the median result of *t*-tests performed on log-transformed PM probe data. ChipStat differs from this approach in several significant respects. These include the use of a fixed *P *value threshold for pairwise probe comparisons and the use of the degree of reproducibility across the entire probe set as an indication of statistical significance. Results from the empirical control data suggest that ChipStat performs slightly better than logit-T in most cases within our biological system. Interestingly, however, the advantage of ChipStat over logit-T was more modest than the advantage over SAM, dChip, and the *t*-test; as logit-T also uses probe-level comparisons, this result is consistent with our overall observations regarding the increased power of probe-based analysis. It is also worth noting that the nominal *P *values derived from both logit-T and dChip substantially underestimated statistical significance prior to correction with our control data, suggesting that, for example, the median *P *value cannot be used to directly assess significance without such correction.

One additional difference between ChipStat and logit-T stems from the use of mismatch (MM) probe cells (ChipStat) and log-transformed data (logit-T). As currently implemented, the ChipStat algorithm compares differences in probe pair (PM - MM) values rather than in PM values alone. Interestingly, the use of PM values within the ChipStat algorithm does not result in superior performance (data not shown), and log(PM) data yield performance that is roughly comparable to PM - MM (data not shown). Further work will be required to determine if the log(PM) approach can be adapted to improve the performance of ChipStat.

The Intersector algorithm tabulates MAS calls from all pairwise comparisons across replicate groups. As we have shown, this algorithm provides the most sensitive method for detecting gene expression changes at low false-detection rates. However, it suffers from several substantial drawbacks. First, the proprietary nature of the Affymetrix algorithm and its associated decision matrices limits the ability to automate the analytical process. Additionally, because N^2 ^pairwise comparisons are required for equal groups of N replicates (that is, *O*(N^2^)), this method is not easily scalable to larger numbers of samples. In contrast, ChipStat scales linearly with N, and the use of the heteroscedastic *t*-test also makes it possible to precompute results for a (potentially large) baseline control population against which multiple comparisons will be performed. While both approaches are feasible for triplicate comparisons, extension of Intersector to much larger numbers is unlikely to be practical.

A third disadvantage to the Intersector approach stems from the lack of a detailed model for its underlying statistical framework. Both ChipStat and Intersector, as currently described, require the use of control samples to generate an estimate of statistical significance. Thus, extension of these results to encompass either a substantially different experimental system or larger numbers of replicates will require the generation of new empirical significance curves. In the case of large numbers of replicates, however, the cost of generating such data is likely to remain prohibitive at least in the near future. Statistical simulations of ChipStat behavior may, however, provide a mechanism for extending the current control curves for larger datasets. This approach would rely on the relative estimates of E(b) + E(p) and E(h) obtained by fitting the current empirical curve derived from six samples. While a small number of probe sets change more often than would be predicted by simulation, it should be possible to conservatively estimate an upper bound to the P value curve by overestimating the relative contribution of E(b) + E(p) vs. E(h). Further experimental work will be required to confirm this possibility.

One caveat to this approach is that the simplified statistical model that we have used to illustrate the theoretical advantages of probe-level comparisons does not account for the variability in the behavior of specific probe cells that exist on Affymetrix arrays. In contrast, the model-based approach to signal estimation implemented in dChip explicitly incorporates such variability and has been shown to provide a good fit to empirical measurements of array data [8,32]. It is likely that incorporation of probe-specific parameters in this way would improve the ability to predict the theoretical behavior of ChipStat and provide a better estimate of hybridization noise from our empirical data. Given this likelihood, current estimates of the relative contributions of E(b) + E(p) and E(h) should be taken as provisional.

In this context, it is worth noting that the ability to perform our current validation and to tune both Intersector and ChipStat was critically dependent on the gene-expression datasets derived from our independent cohorts of control animals. One might naively assume, in the absence of true negative control data, that robust changes in gene expression should be detected by simply taking the Affymetrix MAS calls and requiring that they consistently demonstrate increases for all pairwise comparisons. In contrast, our data show that the Intersector algorithm can achieve increased sensitivity while retaining an appropriately low (and defined) false-positive rate. Both the Intersector and ChipStat algorithms can be tuned using negative control data for sensitivity versus false-positive rate, depending on the type of analysis and application-specific tolerance for false-positive calls. Furthermore, these algorithms can be combined to further improve their sensitivity. As we have demonstrated that each of these algorithms detects a population of probe sets not identified by the other at a comparable stringency, this combined approach may yield the best result. Given these considerations, we favor the use of the hybrid ChipStat/Intersector approach for small number of replicates (around three), with ChipStat alone being useful for large numbers of replicates. Although ChipStat shows greater sensitivity than logit-T at moderate numbers of false positives (more than five expected false positives out of 12,488 probe sets), their comparable performance at high stringency (less than five expected false positives) suggests that the overlap in genes identified by these two techniques may also be of interest.

An additional piece of evidence for the utility of our approach is provided by the statistical association of GO annotation with lists derived from ChipStat/Intersector, dChip or logit-T. At the level of significance tested (3.4 genes per list expected by chance), ChipStat/Intersector lists were statistically associated with a greater number of GO terms than were lists derived from dChip or logit-T. Furthermore, as would be predicted from the fact that logit-T is also a probe pair-level comparison method, logit-T lists are associated with GO terms at a level that is intermediate between dChip and ChipStat/Intersector. One of the terms associated only with the ChipStat/Intersector list of downregulated genes is 'mitochondrial inner membrane'; this example is particularly noteworthy in light of previous work demonstrating a presumptive role for enzymes of fatty acid oxidation in adaptive thermogenesis during the neonatal period [4].

It should be noted that these results depend on the level of significance chosen for generation of the original lists, and an increase in the total number of differentially expressed genes identified may actually decrease the statistical significance of a given association if it does not result in the detection of more genes within the category in question. Despite some caveats as to the generalizability of these results, our data demonstrate that the improved sensitivity of ChipStat/Intersector can measurably influence the ability to interpret patterns of biological activity.

### Early murine mammary gland development

For the FVB murine mammary gland, the period from 2 to 5 weeks of age encompasses critical developmental milestones that include the suckling-weaning transition as well as the profound hormonal changes that characterize the onset of puberty and its consequent rapid ductal epithelial proliferation. Our present work has more completely characterized changes in the transcriptome that occur during early murine mammary gland development than have previous reports. A total of 213 upregulated and 130 downregulated probe sets were identified under conditions designed to yield a low expected false-positive rate (3.4 probe sets expected to change by chance per list).

Four out of five of the most highly upregulated transcripts through the onset of puberty are members of the MUP family of odorant-binding proteins. MUPs are lipocalins that can bind hydrophobic molecules such as pheromones, and they have previously been shown to play a role both in the delivery of signals within the urine as well as in the reception of these signals on the nasal epithelium ([33,34]; see [35] for review). Isoform-specific MUP expression has also previously been reported in a number of secretory glands, including the murine mammary gland [23,24]. However, detectable expression has previously been reported only beginning with the first pregnancy [23].

Our results demonstrate a striking increase in the expression of a variety of MUP isoforms as the mammary gland makes the transition from the neonatal period through the beginning of puberty. This expression pattern is noteworthy given the known effect of puberty on MUP expression in the liver [36]. Interestingly, however, expression in the liver is markedly greater in the male and has been causally linked to the male pattern of growth-hormone pulses [36]. As this male-specific pattern of expression has been shown to be Stat5b-dependent, the availability of Stat5b -/- mice should allow future determination of whether mammary expression is mediated via a similar signaling pathway. Regardless of this, despite an interval of over two decades since the first description of MUP expression in the mammary gland, a functional role has still not been elucidated. Although it has long been assumed that MUP synthesis occurs in the secretory epithelium of expressing organs, our observation that these molecules are upregulated during puberty (with a corresponding approximately threefold downregulation following puberty, data not shown) suggests that their functional role may not be limited to the secretory function of the gland.

Delta-like kinase (Dlk1) is a member of the epidermal growth factor (EGF) superfamily [37] that is encoded on murine chromosome 12 [38]. *Dlk1 *is one of several genes showing substantial (greater than fivefold) downregulation from 2 to 5 weeks of murine mammary gland development. As this gene was first identified as a preadipocyte transcript that is downregulated during subsequent differentiation [38], we hypothesize that its relatively high expression during the neonatal period reflects ongoing differentiation of the mammary fat pad. This kinase has also been shown to have a role in other developmental contexts, specifically within neuroendocrine tissues. Further work will be required to elucidate its specific role in the mammary gland. Notable, however, is the corresponding downregulation (more than 10-fold) of *Meg3/Gtl2*, a noncoding RNA that is reciprocally imprinted with *Dlk1 *[26]. This *Dlk1-Meg3/Gtl2 *regulation has been compared with *Igf2-H19*, another tandem pair of reciprocally imprinted genes in which one member produces a noncoding RNA [27,39]. Interestingly, both *Igf2 *and *H19 *are also downregulated during this time period, suggesting the hypothesis that a common regulatory mechanism exists for the tandem control of both imprinted genes at these loci. It will be particularly important to determine whether there is functional significance to this *Igf2-H19 *regulation, or whether it reflects the epiphenomenal byproduct of a mechanism designed to downregulate *Dlk1 *during adipocyte development.

## Conclusions

We have developed two novel algorithms for the analysis of Affymetrix oligonucleotide microarray data. We have validated these algorithms by using empirically derived distributions from control animals to calibrate their statistical significance. These control data, which reflect both experimental and biological sources of variability likely to be representative of many mammalian experimental systems, should facilitate further work in this area. For triplicate samples, Intersector appears to provide the most sensitivity at a given threshold of statistical significance, and its performance is substantially superior to other widely used methods including the *t*-test, SAM, dChip, and logit-T. However, its lack of scalability, along with the baseline time required for processing, make it unsuitable for larger numbers of replicates. ChipStat, in contrast, provides comparable sensitivity with triplicate samples and has the capability of handling much larger numbers of replicates in order to improve the reliable dectection of small changes in gene expression. Both algorithms provide a substantial increase in the ability to sensitively detect statistically significant changes in gene expression within the context of the whole mammary gland.

We have applied these techniques to the analysis of genomic patterns during early murine mammary gland development. In addition to detecting patterns reflecting known biology, we have noted the coordinate upregulation of a class of molecules not previously known to be differentially regulated in the mammary gland. We also suggest that peri-pubertal changes in the mammary gland may utilize mechanisms for tandem upregulation of multiple imprinted regions. Our observations suggest a variety of future directions for functional validation and demonstrate the utility of coupling sensitive detection of differential gene expression with pathway analysis for the elucidation of biological patterns during organogenesis.

## Materials and methods

### Animals, RNA isolation, and northern blot hybridization

The third, fourth and fifth mammary glands were harvested from FVB mice at the indicated time points. Samples from 2 and 5 weeks of age reflect triplicate pools of 10 animals at each time point (total 60 animals). In addition, tissue from 18 control animals was harvested when they were 6 weeks and 4 days old. These control animals also carry a transgenic construct consisting of the murine mammary tumor virus (MMTV) promoter upstream of the reverse tetracycline transactivator (rtTA) and had been given 2.0 mg/ml doxycycline in drinking water for 96 h before harvest. This line (previously designated MTB) has been previously described, and no developmental abnormalities have been noted [40]. All animal experimentation was conducted in accord with accepted standards of humane care, and protocols for animal work were approved by the University of Pennsylvania institutional committee on animal care.

All tissue was snap frozen after removal of the lymph node present in the fourth gland, and total RNA was isolated by homogenization in guanidinium isothiocyanate and subsequent centrifugation through a cesium chloride cushion as previously described [41]. Northern blot hybridization was performed as previously described [42].

### Arrays and hybridization

Approximately 15-20 *μ*g total RNA was used for each hybridization. RNA was visualized by gel electrophoresis to ensure its integrity before analysis. Biotinylated cRNA was generated and hybridized to Affymetrix MG_U74Av2 arrays according to the manufacturer's instructions. To scale between chips, these expression values were rank ordered, and the median approximately 96% were averaged. Chips were scaled relative to each other to equalize this average value. All Affymetrix control probe sets were eliminated from analysis, yielding data from a total of 12,422 probe sets. Datasets are publicly available as CEL files designated MTB_ [1-6] (Additional data files 3-8 available with the online version of this paper), 2wk_G0P0_ [1-3] (Additional data files 9-11) and 5wk_G0P0_ [1-3] (Additional data files 12-14) containing results derived from control cohorts, 2-week nulliparous cohorts, and 5-week nulliparous cohorts respectively.

### Algorithms and software

To detect differentially regulated genes, we implemented an algorithm (ChipStat) that takes identical probe pairs across two comparison groups and performs a heteroscedastic *t*-test. The number of probe pairs within a probe set that are significantly different (*P *<*p*_ps _where *p*_ps _is a fixed value) was tabulated. We consider that a greater number of probe pairs changing in a given direction indicates a greater probability that the gene detected by the probe set is differentially expressed. If the bulk of the noise within the array data derives from pre-hybridization experimental factors (that is, E(b) + E(p); see Results section for definition), the expectation is that all probe pairs would change coordinately. That is, if there are 16 probe pairs in the probe set, we would expect (for E(b) + E(p) >> E(h)) that under the null hypothesis (no change in gene expression) either 0/16 or 16/16 probe pairs should change significantly (at frequencies of approximately 1 - *p*_ps _and approximately *p*_ps_, respectively). Conversely, if the bulk of the noise derives from hybridization to individual probe cells (that is, if E(b) + E(p) << E(h)), then the number of probe pairs *r *that change within a given probe set of size *t *can be approximated by the binomial distribution:



However, under experimentally realistic conditions, neither of these limiting cases is likely to apply. Therefore, to empirically determine the null distribution using six independent, biologically identical control populations, all pairwise three by three combinations were compared and the number of probe pairs changing was tabulated. To determine the expected number of changes per probe set when fewer than 16 probe pairs are available, these analyses were repeated after randomly discarding 1, 2...15 probe pairs. In this way, a similar statistical estimate was obtained for the 602 probe sets on the MG_U74Av2 array that have fewer than 16 probe pairs per probe set. A conservative simplification of these data was performed by rounding up the significance of changes in these 602 probe sets to the nearest appropriate bin in the 16 probe pair per probe set curve. A Microsoft Windows-compatible application implementing the ChipStat algorithm is freely available for academic use [43].

On the basis of the simplified statistical model described, a Monte Carlo simulation was implemented to determine the number of expected false-positive values as a function of *p*_ps _for various relative proportions of E(b) + E(p) and E(h). Briefly, a random test dataset was generated in which equal gene expression was perturbed by Gaussian noise (representing E(b) + E(p)). Each expression value was then independently perturbed 16 times (representing 16 probe pairs/probe set) by another Gaussian noise function (representing E(h)), and comparisons were tabulated using the ChipStat algorithm. This simulation was implemented in C and the source code is available [43]. All values reported reflect the mean of 100 trials, where each trial simulates 11,820 probe sets with 16 probe pairs each. The relative contributions of E(b) + E(p) and E(h) were estimated by maximizing the likelihood function:



with respect to (E(b) + E(p)) / E(h) where *x*_*i *_is the number of times *i *probe pairs increased significantly and *μ*_*i *_and *σ*_*i *_represent the mean and standard deviation from the Monte Carlo simulations.

A separate algorithm (Intersector) uses pairwise calls of differential gene expression derived from Affymetrix Microarray Suite (MAS) analysis. All pairwise comparisons were performed (that is, 3 × 3 = 9 comparisons for a 3- vs 3-replicate comparison) using the manufacturer's default settings, and the number of 'increases' or 'marginal increases' was tabulated. Similarly to the ChipStat method described above, the null distribution was generated by tabulating results from all 20 distinguishable 3 vs 3 combinations of the six control samples. Results were obtained using both MAS version 4 (MAS4) and MAS version 5 (MAS5), as indicated in the text and figures.

Tests for differential gene expression using a homoscedastic *t*-test or SAM [11] were performed using signal values derived from MAS5. SAM results were obtained using software obtained from its authors [44]. Because the analyses described are reported as a function of the number of genes expected to increase by chance (essentially a one-tailed test of significance), the false-discovery rate reported by SAM was multiplied by 0.5 to derive a corrected false-positive rate (false-increase rate). dChip analysis [8] was performed using software available from its authors [45], and a PM-only expression model was constructed. Logit-T analysis [10] was performed using software provided by its authors and compiled to run locally on an AMD Linux server. Both dChip and Logit-T significance values were empirically calibrated by analyzing all possible 3 vs 3 combinations of control arrays (20 total) and tabulating the average number of false positives as a function of the reported significance.

### Association with biological annotation

Associations between GO [19] annotation and lists of differentially expressed genes were identified using EASE [20]. Multiple testing correction was performed using within-system bootstrapping, and a final cutoff of *P *< 0.05 was used to identify statistically significant associations.

## Additional data files

The following additional data are available with the online version of this paper. Additional data file 1 contains a table showing ChipStat and Intersector in combination. For each level of stringency available, the pairwise intersection of ChipStat (CS, *p*_ps _= 0.05) and Intersector (IT, MAS5) lists of significantly increasing probe sets was generated. Rows indicate the threshold number of probe pairs (0-16) significantly increasing from ChipStat, and columns indicate the threshold number of Increase or Marginal Increase calls (0-9) identified by Intersector. (**a**) Number of increasing probe sets in 2- vs 5-week murine mammary gland. Selected results correspond to values plotted on the *y *axis of Figure 4d (number of probe sets increasing). (**b**) Average number of increasing probe sets using all 3 × 3 combinations of 6 negative control samples. Selected results correspond to values plotted on the *x *axis of Figure 4d (expected number of probe sets increasing by chance). Additional data file 2 contains a table showing differential gene expression in 2- vs 5-week murine mammary gland using a hybrid ChipStat/Intersector approach. The criteria ChipStat *p*_ps _= 0.05, 6/16 probe pairs increasing and Intersector 7/9 increases + marginal increases, were used to identify lists of probe sets that are up- and downregulated from 2 to 5 weeks of FVB female murine mammary gland development. Additional data files 3,4,5,6,7 and 8 contain six control files containing CEL file data from Affymetrix MG_U74Av2 oligonucleotide microarrays hybridized to RNA from the third to fifth mammary glands harvested from independent pools of three female MTB transgenic mice at 6 weeks 4 days old after 96 hours of doxycycline treatment. Additional data files 9,10 and 11 contain three CEL files of data from Affymetrix MG_U74Av2 oligonucleotide microarrays hybridized with mammary gland RNA from independent pools of 10 female FVB mice harvested at 2 weeks of age. Additional data files 12,13 and 14 contain three CEL files of data from Affymetrix MG_U74Av2 oligonucleotide microarrays hybridized to mammary gland RNA from independent pools of 10 female FVB mice harvested at 5 weeks of age.

## Supplementary Material

Additional data file 1 A table showing ChipStat and Intersector in combination Click here for additional data file

Additional data file 2 A table showing differential gene expression in 2- vs 5-week murine mammary gland using a hybrid ChipStat/Intersector approach Click here for additional data file

Additional data file 3 Control file 1 containing CEL data from Affymetrix MG_U74Av2 oligonucleotide microarrays hybridized to RNA from the third to fifth mammary glands harvested from independent pools of three female MTB transgenic mice at 6 weeks 4 days old after 96 hours of doxycycline treatment Click here for additional data file

Additional data file 4 Control file 2 containing CEL data from Affymetrix MG_U74Av2 oligonucleotide microarrays hybridized to RNA from the third to fifth mammary glands harvested from independent pools of three female MTB transgenic mice at 6 weeks 4 days old after 96 hours of doxycycline treatment Click here for additional data file

Additional data file 5 Control file 3 containing CEL data from Affymetrix MG_U74Av2 oligonucleotide microarrays hybridized to RNA from the third to fifth mammary glands harvested from independent pools of three female MTB transgenic mice at 6 weeks 4 days old after 96 hours of doxycycline treatment Click here for additional data file

Additional data file 6 Control file 4 containing CEL data from Affymetrix MG_U74Av2 oligonucleotide microarrays hybridized to RNA from the third to fifth mammary glands harvested from independent pools of three female MTB transgenic mice at 6 weeks 4 days old after 96 hours of doxycycline treatment Click here for additional data file

Additional data file 7 Control file 5 containing CEL data from Affymetrix MG_U74Av2 oligonucleotide microarrays hybridized to RNA from the third to fifth mammary glands harvested from independent pools of three female MTB transgenic mice at 6 weeks 4 days old after 96 hours of doxycycline treatment Click here for additional data file

Additional data file 8 Control file 6 containing CEL data from Affymetrix MG_U74Av2 oligonucleotide microarrays hybridized to RNA from the third to fifth mammary glands harvested from independent pools of three female MTB transgenic mice at 6 weeks 4 days old after 96 hours of doxycycline treatment Click here for additional data file

Additional data file 9 CEL file 1 of data from Affymetrix MG_U74Av2 oligonucleotide microarrays hybridized with mammary gland RNA from independent pools of 10 female FVB mice harvested at 2 weeks of age Click here for additional data file

Additional data file 10 CEL file 2 of data from Affymetrix MG_U74Av2 oligonucleotide microarrays hybridized with mammary gland RNA from independent pools of 10 female FVB mice harvested at 2 weeks of age Click here for additional data file

Additional data file 11 CEL file 3 of data from Affymetrix MG_U74Av2 oligonucleotide microarrays hybridized with mammary gland RNA from independent pools of 10 female FVB mice harvested at 2 weeks of age Click here for additional data file

Additional data file 12 CEL file 1 of data from Affymetrix MG_U74Av2 oligonucleotide microarrays hybridized to mammary gland RNA from independent pools of 10 female FVB mice harvested at 5 weeks of age Click here for additional data file

Additional data file 13 CEL file 2 of data from Affymetrix MG_U74Av2 oligonucleotide microarrays hybridized to mammary gland RNA from independent pools of 10 female FVB mice harvested at 5 weeks of age Click here for additional data file

Additional data file 14 CEL file 3 of data from Affymetrix MG_U74Av2 oligonucleotide microarrays hybridized to mammary gland RNA from independent pools of 10 female FVB mice harvested at 5 weeks of age Click here for additional data file

## Figures and Tables

**Table 1:** Northern blot validation of differential gene expression

Probe set ID	Accession number	Gene	Fold change	Probe pairs increasing	Differential expression confirmed
99067_at	X59846	*Gas6*	3.41	16/16	x
100064_f_at	M63801	*Gja1*	1.67	12/16	x
102016_at	M61737	*Fsp27*	2.07	11/16	x
93996_at	X01026	*Cyp2e1*	11.6	10/16	x
97507_at	X67809	*Ppicap*	2.85	9/16	x
101995_at	U40930	*Sqstm1*	1.48	8/16	x
93096_at	AA986050	*3010002H13Rik*	2.65	7/16	x
102791_at	U22033	*Psmb8*	1.65	7/16	x
96072_at	M17516	*Ldh1*	1.37	6/16	

Genes identified as being differentially expressed were randomly chosen for verification by northern blot hybridization (see text for description). Gene identifiers are shown along with fold changes, numbers of probe pairs increasing (as identified by ChipStat with *p*_ps _= 0.04), and confirmation of differential expression.

**Table 2:** Association with GO annotation

System	Gene category	CS	LT	DC
**(a) Upregulated genes**				
GO Biological Process	Defense response	x	x	x
GO Cellular Component	Extracellular space	x	x	
GO Cellular Component	Extracellular	x	x	
GO Biological Process	Response to biotic stimulus	x	x	x
GO Biological Process	Immune response	x	x	x
GO Biological Process	Response to external stimulus	x	x	x
GO Biological Process	Organismal physiological process	x	x	x
GO Biological Process	Antigen presentation	x	x	
GO Biological Process	Response to stimulus	x	x	x
GO Biological Process	Antigen presentation\, endogenous antigen	x		
GO Molecular Function	MHC class I receptor activity	x		
GO Biological Process	Antigen processing	x	x	
GO Biological Process	Complement activation	x	x	
GO Biological Process	Antigen processing, endogenous antigen via MHC class I	x		
GO Biological Process	Response to pest/pathogen/parasite	x	x	x
GO Biological Process	Humoral defense mechanism (sensu Vertebrata)	x		
GO Molecular Function	Pheromone binding	x	x	
GO Molecular Function	Oxidoreductase activity	x		
GO Molecular Function	Oxidoreductase activity, acting on the aldehyde or oxo group of donors	x		
GO Molecular Function	Odorant binding	x	x	
GO Molecular Function	Transmembrane receptor activity	x		
GO Biological Process	Humoral immune response	x		
				
**(b) Downregulated genes**				
GO Cellular Component	Mitochondrion	x	x	
GO Biological Process	Main pathways of carbohydrate metabolism	x	x	
GO Biological Process	Tricarboxylic acid cycle	x	x	
GO Biological Process	Energy derivation by oxidation of organic compounds	x	x	
GO Biological Process	Energy pathways	x	x	
GO Cellular Component	Mitochondrial membrane	x		
GO Biological Process	Carbohydrate metabolism	x	x	
GO Cellular Component	Inner membrane	x		
GO Biological Process	Blood vessel development	x		
GO Cellular Component	Mitochondrial inner membrane	x		
GO Cellular Component	Extracellular		x	

Lists of differentially expressed genes derived from a hybrid ChipStat/Intersector approach (ChipStat: *p*_ps _= 0.05, 6/16 probe pairs increasing AND Intersector: 7/9 increases + marginal increases), logit-T, and dChip were associated with GO terms using EASE [20]. Individual terms are annotated according to whether association with the given annotation group was statistically significant (*P *< 0.05 using within-system bootstrap to account for multiple testing) using lists derived from ChipStat/Intersector (CS), logit-T (LT), or dChip (DC). **(a) **Association with lists of upregulated genes. (**b**) Association with lists of downregulated genes.
